# Noninvasive pulmonary artery wave intensity analysis in pulmonary hypertension

**DOI:** 10.1152/ajpheart.00480.2014

**Published:** 2015-02-06

**Authors:** Michael A. Quail, Daniel S. Knight, Jennifer A. Steeden, Liesbeth Taelman, Shahin Moledina, Andrew M. Taylor, Patrick Segers, Gerry J. Coghlan, Vivek Muthurangu

**Affiliations:** ^1^Centre for Cardiovascular Imaging, Institute of Cardiovascular Science, University College London and Great Ormond Street Hospital for Children, London, United Kingdom;; ^2^Department of Cardiology, Royal Free London National Health Services Foundation Trust, London, United Kingdom; and; ^3^IBiTech-bioMMeda, iMinds Medical IT, Ghent University, Gent, Belgium

**Keywords:** pulmonary hypertension, hemodynamics, wave intensity, cardiac magnetic resonance imaging

## Abstract

Pulmonary wave reflections are a potential hemodynamic biomarker for pulmonary hypertension (PH) and can be analyzed using wave intensity analysis (WIA). In this study we used pulmonary vessel area and flow obtained using cardiac magnetic resonance (CMR) to implement WIA noninvasively. We hypothesized that this method could detect differences in reflections in PH patients compared with healthy controls and could also differentiate certain PH subtypes. Twenty patients with PH (35% CTEPH and 75% female) and 10 healthy controls (60% female) were recruited. Right and left pulmonary artery (LPA and RPA) flow and area curves were acquired using self-gated golden-angle, spiral, phase-contrast CMR with a 10.5-ms temporal resolution. These data were used to perform WIA on patients and controls. The presence of a proximal clot in CTEPH patients was determined from contemporaneous computed tomography/angiographic data. A backwards-traveling compression wave (BCW) was present in both LPA and RPA of all PH patients but was absent in all controls (*P* = 6e^−8^). The area under the BCW was associated with a sensitivity of 100% [95% confidence interval (CI) 63–100%] and specificity of 91% (95% CI 75–98%) for the presence of a clot in the proximal PAs of patients with CTEPH. In conclusion, WIA metrics were significantly different between patients and controls; in particular, the presence of an early BCW was specifically associated with PH. The magnitude of the area under the BCW showed discriminatory capacity for the presence of proximal PA clot in patients with CTEPH. We believe that these results demonstrate that WIA could be used in the noninvasive assessment of PH.

pulmonary hypertension (PH) is primarily characterized by increased pulmonary vascular resistance (PVR) and reduced pulmonary arterial (PA) compliance (15, 31). However, arterial wave reflections, which are caused by abrupt changes in vessel area or compliance, also contribute to right ventricular (RV) load. As PH is characterized by widespread vascular changes, it has been postulated that abnormal wave reflections may be an additional source of increased afterload.

For this reason, assessment of wave reflections maybe clinically useful in PH and could be achieved using wave intensity analysis (WIA). Proposed by Parker and Jones (24), WIA allow assessment of the type (compression or expansion), direction (forward or backward), intensity, and timing of vascular waves (37). Importantly, WIA provides information about the vasculature that cannot be evaluated using conventional measures of PVR and compliance. With the use of WIA, it has been shown that pathological wave reflections are present in animal models of PH and in PH patients (9, 16). Unfortunately, conventional WIA requires invasive measurement of pressure and velocity, which hinders its use in the clinical environment (9).

Recently, it has been demonstrated that WIA can be performed noninvasively using image-based measures of arterial distension and blood flow (1, 6). Although the initial implementation by Feng and Khir relied on ultrasound imaging (6), phase contrast magnetic resonance (PCMR) can also provide accurate measures of distension and flow (17). However, high temporal resolution data are necessary to accurately assess wave speed (an important step in WIA) (27) and this conventionally requires long free breathing acquisitions. Unfortunately, images acquired during free breathing acquisitions provide inadequate vessel wall delineation for WIA.

One solution is to highly accelerate the acquisition to enable high temporal resolution data to be acquired in a breath hold as proposed by Biglino et al. (1). This approach has proven successful in relatively fit adults but may be difficult to apply in PH patients who often find breath holding difficult. An alternative solution is respiratory navigated PCMR, which should allow acquisition of high temporal resolution images without corruption of the vessel edge by breathing artifacts.

In this study, we used a previously validated respiratory self-navigated golden angle spiral PCMR sequence to acquire vessel area and flow data to perform WIA in volunteers and patients with PH. This sequence provides data with a sampling frequency three to four times higher than conventional CMR flow imaging and overcomes the problems of vessel wall blurring due to breathing.

The aims of this study were *1*) to demonstrate the feasibility of noninvasive pulmonary WIA; *2*) characterize patients with PH and healthy controls; *3*) assess the ability of WIA to discriminate patients with proximal chronic thromboembolic pulmonary hypertension (CTEPH) disease from other forms of PH; and *4*) evaluate the relationship between abnormal wave reflections and steady-state hemodynamics.

## METHODS

### Subjects

Twenty consecutive patients with PH undergoing right heart catheterization and 10 healthy volunteers were recruited. PH was diagnosed by right heart catheterization as a mean PA pressure >25 mmHg and a pulmonary capillary wedge pressure <15 mmHg (7). Exclusion criteria were *1*) irregular heart rates; *2*) contraindications to cardiovascular magnetic resonance (CMR) such as MR-incompatible implants; *3*) known independent left-sided cardiac disease unrelated to PA hypertension; *4*) clinically significant restrictive or obstructive lung disease identified by pulmonary function tests; or *5*) pregnancy. The study was performed with local research ethics committee approval and written informed consent was obtained.

### MR Protocol

All imaging was performed on a 1.5T CMR scanner (Magnetom Avanto; Siemens Healthcare, Erlangen, Germany), using two rows of spine coil elements and two rows of body-matrix elements, giving a total of 12 coil elements. A vector electrocardiographic system was used for cardiac gating. All flow imaging for WIA was performed using a self-navigated, cardiac gated, golden-angle spiral PCMR sequence (34). In brief, an image-based navigator was first produced by reconstructing low temporal resolution (315 ms) real-time images by combining the data in consecutive groups of 30 spiral pairs. This navigator was then used to select the spiral interleaves acquired in expiration for final reconstruction of the retrospectively cardiac-gated data. Sequence parameters were as follows: echo time (TE)/repetition time (TR) 2.7/5.26 ms; field of view (FOV) 450 × 450 mm; matrix: 192 × 192; uniformly distributed spiral interleaves required to fill k-space: 80; slice thickness: 7 mm; velocity encoding gradient (VENC): 150 cm/s; flip angle: 25°; and pixel bandwidth: 1, 628 Hz/pixel. This achieved a temporal resolution of 10.5 ms, with a spatial resolution 2.34 × 2.34 mm, giving ∼90 cardiac phases, in a scan time of ∼4 min. PA flow imaging was performed at the approximate midway point of both the left and right PAs. The right and left PAs were used to avoid the through plane motion of the pulmonary trunk, and to facilitate the investigation of asymmetric lung involvement.

### Image Processing

All images were processed using an in-house plug-in for the open source DICOM software OsiriX (OsiriX Foundation, Geneva, Switzerland) (29). Segmentation of the branch PAs was performed on the modulus image using a previously validated semiautomatic registration-based algorithm (22). The branch PA region of interest (ROI) could also be manually altered to ensure optimal vessel wall delineation. The final ROI was used to both calculate the cross-sectional area (A) and prescribe the region in the phase image from which flow (Q) was calculated.

### Signal Processing

For wave speed analysis, A and Q curves were not interpolated or filtered. For WIA the A and Q curves were interpolated to 1-ms temporal resolution using a cubic spine and filtered using a zero-phase, low-pass, 2nd order Butterworth filter with cut-off frequency of 20 Hz. All signal processing was performed in Matlab 2012a (Mathworks).

#### Wave speed calculation.

Wave speed (*c*) was calculated using the QA method (28, 36), as deduced from the water hammer equations. Wave speed is equivalent to pulse wave velocity (PWV). This method relies on the fact that:

(1) $$c=\pm \frac{dQ_{\pm }}{dA_{\pm }}$$

in the presumably reflection free part of early systole (with *c* in m/s, dQ in m^3^/s, and dA in m^2^). In our implementation, the gradient of Q against A was calculated by linearly regressing the first three unfiltered and uninterpolated points of the Q and A curves at the start of systole (26). Only the first three points (first ∼30 ms of systole) were used to ensure that there was minimal signal contamination from wave reflections (35).

#### Wave intensity analysis.

In WIA, waves are regarded as a summation of incremental wave fronts; it is therefore possible to separate the Q and A curves into the respective forward (+) and backward (−) components by expressing the relationship between *c*, and changes in flow and cross-sectional area. *Equation 1* combined with *Eqs. 2* and *3*:

(2) $$dA=dA_{+}+dA_{-}$$

(3) $$dQ=dQ_{+}+dQ_{-}$$

can be solved for the changes in the forward and backward flow and cross-sectional area; this results in *Eqs. 4* and *5*:

(4) $$dQ_{\pm }=\frac{1}{2}\left(dQ\pm cdA\right)$$

(5) $$dA_{\pm }=\frac{1}{2}\left(A\pm \frac{1}{c}dQ\right)$$

Net wave intensity d*I*_a_ was defined as the product of the differentials of cross-sectional area and flow.

(6) $$dl_{a}=dA\;dQ$$

Similarly, it can be shown that the net wave intensity d*I*_a_ (*Eq. 6*) can be divided into the forward and backward intensities, *Eq. 7*:

(7) $$dl_{a}=dl_{a\left(+\right)\;}+\;dl_{a\left(-\right)}$$

with the separated d*I*_a_ expressed as:

(8) $$dl_{a\left(\pm \right)}=\pm \frac{c}{4}{\left[dA\pm \frac{dQ}{c}\right]}^{2}$$

With the use of this formulation forwards and backwards d*I*_a_ were calculated and plotted. As per convention, the direction of waves was referenced to the direction of blood flow. Waves arising from the heart were defined as forward running and those arising from the vasculature as backward running. Waves causing an increase in area were classified as compression waves and those causing a decrease in area as expansion waves by examination of dA_±_ plots. Thus a forward running wave was held to be a compression wave if dA_+_ was greater than zero and an expansion wave if dA_+_ was less than zero. Similarly, a backward-running wave was considered as a compression wave if dA_−_ was greater than zero and an expansion wave if dA_−_ was less than zero.

Using this system we characterized three different early to midsystolic (flow onset to flow peak) waves: forwards compression waves (FCW), backwards compression waves (BCW), and backwards expansion waves (BEW).

#### WIA postprocessing.

The type, duration, magnitude and time to peak (time from onset of ejection to waveform peak) of waves were determined by analysis of the net and separated WIA plots in Matlab. The areas under the separated waveforms were calculated by numerical integration.

As well as separate quantification of magnitude, timing, and waveform areas: the average of all WIA metrics of both branch PAs was also calculated (FCW_mean_, BCW_mean_, BEW_mean_, and PWV_mean_).

### Catheterization and Clinical Data

Right heart catheterization was performed in all patients with PH according to standard procedures, within 30 days of MR imaging using a Swan-Ganz catheter. Cardiac output was measured using thermodilution. Systolic (SPAP), diastolic (DPAP), and mean (MPAP) PA pressures, pulmonary capillary wedge pressure, and PVR data were obtained. All patients had serum NH_2_-terminal pro-brain natriuretic peptide (NT-pro-BNP) levels, and a 6-min walk test was measured. The clinical subtype of PH was determined by review of patient records. For patients with CTEPH, the presence and location of proximal clot were determined and differentiated from distal disease by review of contemporaneous computed tomography and selective digital subtraction angiographic clinical data. RV ejection fraction (RVEF) was calculated as previously described (18) from a RV transaxial stack using a radial k-t SENSE real-time sequence. The branch PA flow ratio was used to assess any asymmetry in lung blood flow; it was calculated by dividing branch PA flow by total pulmonary blood flow. Acceleration times for both branch PAs and their average were calculated as the time from the onset of ejection to peak flow.

### Statistics

STATA 13 and Graphpad Prism 5f for mac were used for statistical analysis and Figs. Data were examined for normality using the Shapiro-Wilk normality test. Descriptive statistics are expressed as means ± SE when normally distributed and median [interquartile range (IQR)] when nonnormally distributed. Proportions are expressed as percentages.

Pearson's correlation coefficient was used to analyze simple linear relationships between variables. The independent samples *t*-test was used to compare differences in parametric data between PH patients and controls; Welch's correction was employed for unequal variances. The Mann-Whitney *U*-test was used for nonparametric data. Fisher's exact test was used to compare proportions data.

Stepwise binary logistic regression analysis was used to identify covariates with independent association with the diagnosis of PH.

Area under the receiver operating characteristics curve was used to assess the diagnostic accuracy of WIA metrics for the identification of proximal clot in patients with CTEPH. For this analysis, the 40 branch PAs (left and right in 20 PH patients) were coded according to the presence (8 lungs) or absence (32 lungs) of proximal clot on contemporaneous computed tomography or angiographic imaging. The optimum cut-off value was chosen to maximize the Youden index (sensitivity + specificity −1).

Multivariable linear regression analysis was used to determine covariates independently associated with transpulmonary gradient (TPG) and PVR. *P* < 0.05 was considered statistically significant.

## RESULTS

### Study Population Characteristics

Mean age of PH patients was 54 ± 3 yr (15 female, 5 male) and mean age of controls was 47 ± 3 yr (6 female, 4 male); there was no significant difference in age or gender between groups.

The diagnoses in the patient group were as follows: eight systemic sclerosis (7 limited cutaneous, 1 diffuse cutaneous), seven CTEPH, two systemic lupus erythematosus (SLE), two mixed connective tissue disease, and one idiopathic PA hypertension.

The median interval between right heart catheterization and CMR was 6 days (IQR 2–11 days). Hemodynamic data were available for all patients. Mean MPAP was 43 ± 3 mmHg, SPAP was 70 ± 5 mmHg, and DPAP was 27 ± 2 mmHg. Mean pulmonary capillary wedge pressure was 11 ± 1 mmHg and mean PVR was 7.4 ± 0.8 Wood units (WU).

In patients the mean 6-min walk test was 338 ± 28 m and median serum NT-pro-BNP was 145 pmol/l (IQR 225 pmol/l). PH mean CMR RVEF was 41 ± 3%.

In the CTEPH group, five out of seven patients had proximal disease (2 right lobe and 3 bilateral). There was no significant difference between CTEPH patients with proximal disease (*n* = 5) and other PH patients (*n* = 15, other PH etiologies) based on cardiac catheterization data: PVR (*P* = 0.4), MPAP/TPG (*P* = 1.0/*P* = 0.7), pulse pressure (*P* = 0.6), or clinical parameters: 6-min walk test (*P* = 0.9), serum NT-pro-BNP (*P* = 0.8), and CMR RVEF (*P* = 0.9).

### WIA in Patients and Controls

The PWV was approximately two times higher in patients compared with controls Table 1 (PWV_mean_: 1.36 ± 0.08 vs. 0.72 ± 0.05 m/s, *P* = 3e^−7^).

**Table 1.** Comparison of WIA metrics between PH patients and controls

Parameter	Patient	Control	*P*
PWV,‡ m/s			
Right	1.26 (0.07)	0.73 (0.07)	4e^−5^
Left	1.46 (0.12)	0.70 (0.06)	1e^−5^
Mean	1.36 (0.08)	0.72 (0.05)	3e^−7^
Acceleration time,‡ ms			
Right	60 (4)	108 (9)	0.0004
Left	74 (5)	135 (16)	0.005
Mean	67 (4)	121 (11)	0.001
FCW peak,† cm^5^/s			
Right	0.09 (0.11)	0.18 (0.14)	0.03
Left	0.06 (0.06)	0.06 (0.08)	0.4
Mean	0.08 (0.08)	0.15 (0.11)	0.06
FCW peak time,‡ ms			
Right	29 (2)	33 (3)	0.2
Left	33 (2)	42 (4)	0.05
Mean	31 (2)	38 (3)	0.03
FCW area,† cm^5^			
Right	0.003 (0.004)	0.005 (0.005)	0.01
Left	0.002 (0.002)	0.003 (0.003)	0.02
Mean	0.002 (0.002)	0.005 (0.003)	0.006
BCW peak,† cm^5^/s			
Right	0.01 (0.02)	0 (0)	6e^−8^
Left	0.006 (0.02)	0 (0)	6e^−8^
Mean	0.01 (0.01)	0 (0)	6e^−8^
BCW peak time,*‡ ms			
Right	73 (6)	—	—
Left	79 (8)	—	—
Mean	76 (6)	—	—
BCW area,† cm^5^			
Right	0.0004 (0.0005)	0 (0)	6e^−8^
Left	0.0003 (0.0006)	0 (0)	6e^−8^
Mean	0.0004 (0.0006)	0 (0)	6e^−8^
BEW peak,† cm^5^/s			
Right	0 (0)	0.02 (0.02)	9e^−6^
Left	0 (0)	0.01 (0.01)	1e^−6^
Mean	0 (0)	0.01 (0.01)	1e^−6^
BEW peak time,*‡ ms			
Right	—	30 (4)	—
Left	40 (2)	56 (7)	—
Mean	41 (2)	45 (5)	—
BEW area,† cm^5^			
Right	0 (0)	0.0005 (0.0008)	9e^−6^
Left	0 (0)	0.0004 (0.0002)	3e^−7^
Mean	0 (0)	0.0005 (0.0005)	1e^−7^

WIA, wave intensity analysis; PH, pulmonary hypertension; PWV, pulse wave velocity; FCW, forward compression wave; BCW, backward compression wave; BEW, backward expansion wave.

* Waveform absent in the majority of 1 group; therefore, statistical testing of timing parameters or ratios was not performed.

† Nonnormally distributed, median (interquartile range), Mann-Whitney-*U*-test.

‡ Normally distributed, mean (SE), *t*-test ± Welch correction for unequal variances.

**Fig. 1.**
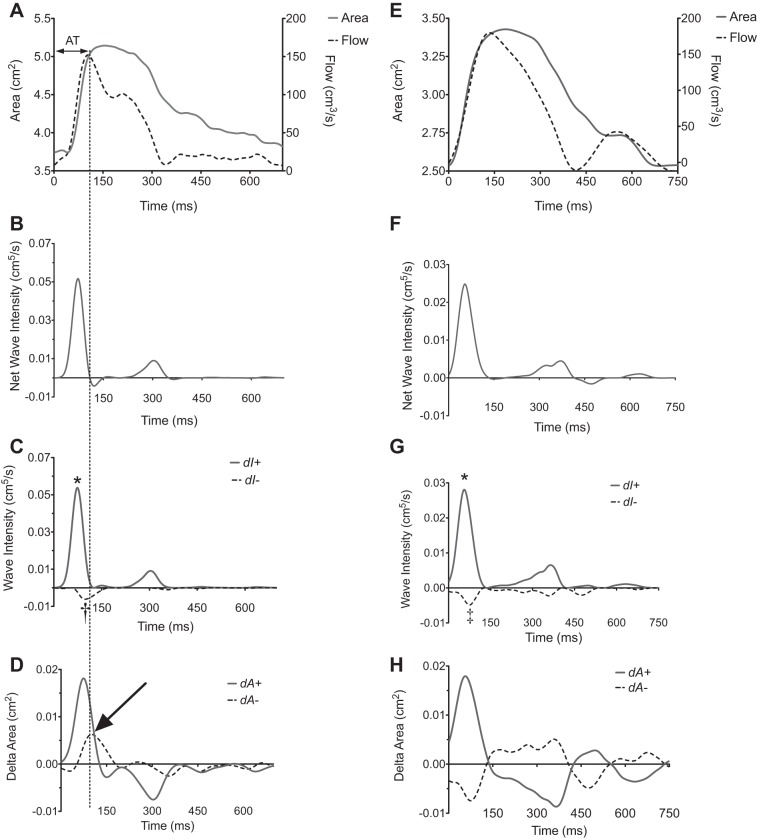
Wave intensity analysis (WIA) in representative pulmonary hypertension (PH) patient (*A*–*D*) and control (*E*–*H*). Three types of waveforms were found to arise during early and mid systole in study participants using wave separation analysis: *1*) a forward compression wave: characterized by increasing area and increasing flow representing cardiac ejection (* in *C* and *G*); *2*) a backwards compression wave: increasing area [pressure] and decreasing flow († in *C*); and *3*) backwards expansion wave: decreasing area [pressure] and/or increasing flow (‡ in *G*). The identification of the backwards compression and expansion waves can be seen from examination of *D* and *H*, showing the dA ± plots. The dotted line across *A*–*D* shows the timing of peak flow used to measure acceleration time (AT), demonstrating it arises as a consequence of the arrival of the backwards compression wave overcoming the forward compression wave (arrow). Time = 0 corresponds to the onset of data acquisition as triggered by the R wave on cardiac magnetic resonance (CMR) vectorcardiography.

Early- and midsystolic forward and backward waves were often found to be coincident on net wave intensity, d*I*_a_ (Fig. 1). Following wave separation into forward and backward components, important group differences were apparent (Fig. 1). Peak wave intensity (cm^5^/s), time to peak wave intensity (ms), and wave intensity area (cm^5^) for FCW, BCW, and BEW are described in Table 1.

**Fig. 2.**
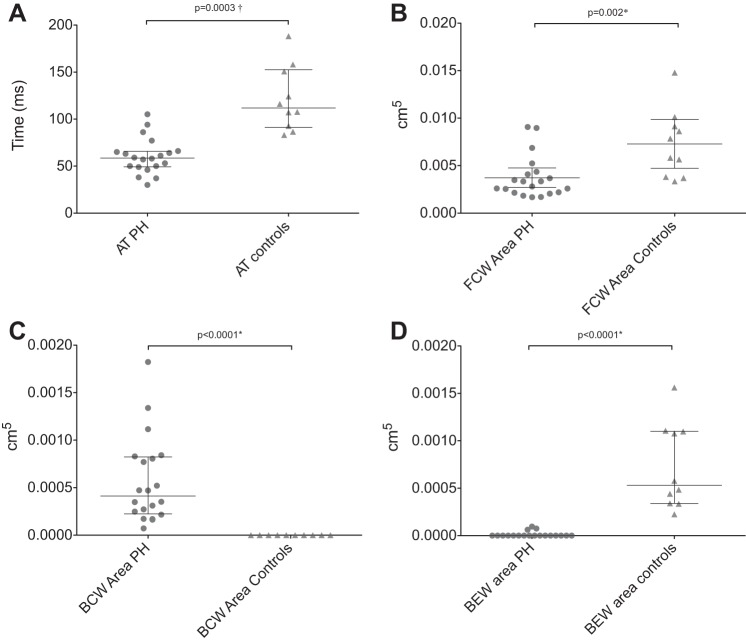
Scatter diagrams of WIA metrics in patients and controls. *A*: mean acceleration time (AT_mean_). *B*: mean forward compression wave (FCW_mean_) area. *C*: mean backward compression wave (BCW_mean_) area. *D*: mean backward expansion wave (BEW_mean_) area.

FCW area was significantly lower in patients than controls as shown in Fig. 2 and Table 1 (FCW_mean_: 0.003 cm^5^ [IQR 0.002] vs. 0.006 cm^5^ [IQR 0.005], *P* = 0.002). The time to peak FCW_mean_ was earlier in patients with PH (mean 31 ± 2 ms compared with controls 38 ± 1 ms, *P* = 0.03).

A BCW was present in both the LPA and RPA of all PH patients but was absent in all controls [*P* = 6e^−8^, odds ratio (OR): 861 [15-∞]; Fig. 2]. Patients' median BCW_mean_ area was 0.0004 cm^5^ (IQR 0.0006 cm^5^), and the time to peak BCW_mean_ was 76 ± 6 ms.

A BEW was present in the RPA of 9/10 controls but was absent in all patients (*P* < 0.0001, OR: 260 [10–6988]). A BEW was present in the LPA of all controls but only in 4/20 of PH patients (*P* < 0.0001, OR: 77 [4–1583]). The median control BEW_mean_ area was 0.0005c m^5^ (IQR 0.0008 cm^5^; Fig. 2). The mean time to peak BEW_mean_ was 45 ± 5 ms.

Acceleration time was significantly lower in patients than controls as shown in Fig. 2 and Table 1 (AT_mean_: 67 ± 4 vs. 121 ± 11 m/s, *P* = 0.001). However, there was still overlap between the two groups. The phenomenon of reduced acceleration time in PH was found to arise as a consequence of the interaction between the FCW and the timing and magnitude of the reflected BCW (Fig. 1).

Stepwise binary logistic regression analysis of variables listed in Table 1 identified the presence of a backward compression wave as the covariate most strongly associated with the presence of PH, discriminating groups completely (−2 log-likelihood: 0).

### PH Subtype Differentiation

**Fig. 3.**
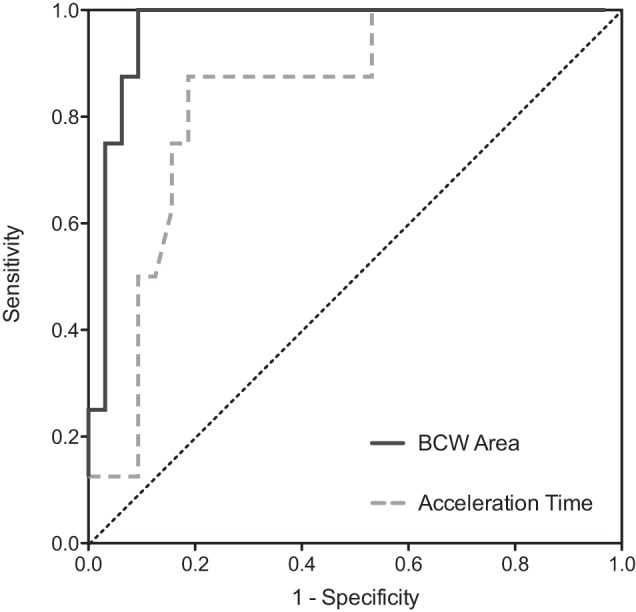
Receiver operating characteristics analysis for the detection of proximal pulmonary artery (PA) clot: sensitivity (*y*-axis) and 1-specificity (*x*-axis). BCW area (black solid line) area under the curve (AUC): 0.97; AT (gray dashed line) AUC: 0.84. Interrupted black line: line of identity.

BCW area and AT showed statistically significant discriminatory capacity for the presence of clot (Fig. 3 and Table 2); PWV, FCW area, FCW peak time, BCW peak time, and branch PA flow ratio were nonsignificant (Table 2).

**Table 2.** Receiver operating characteristics analysis for the detection of proximal PA clot

Variable	AUC	AUC (95% CI)	*P*	Threshold	Sensitivity (95% CI)	Specificity (95% CI)
BCW area	0.97*	0.92–1.0*	0.00005*	>0.0006 cm^5^*	100% (63–100)*	91% (75–98)*
Acceleration time	0.84*	0.70–0.98*	0.003*	<57.6 ms*	88% (47–99)*	81% (64–93)*
PWV	0.70	0.47–0.92	0.09	—	—	—
FCW area	0.61	0.38–0.85	0.3	—	—	—
BCW peak time	0.60	0.40–0.79	0.4	—	—	—
FCW peak time	0.50	0.27–0.74	1.0	—	—	—
Branch PA flow ratio	0.63	0.40–0.87	0.3	—	—	—

PA, pulmonary artery; AUC, area under the receiver operating characteristic curve; CI, confidence interval.

* Significant parameters.

**Fig. 4.**
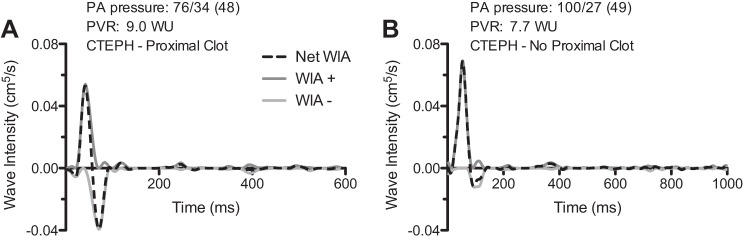
Right PA WIA in 2 patients with chronic thromboembolic pulmonary hypertension (CTEPH). Patient with proximal clot in right lower lobe artery (*A*) and patient with disease limited to distal vessels. Note larger BCW in *patient A* (*B*). PVR, pulmonary vascular resistance; WU, wood units.

The area under the curve (AUC) for BCW area was 0.97 [95% confidence interval (CI) 0.91–1.0]; *P* = 0.00006. A BCW area threshold of >0.0006 cm^5^ was associated with a sensitivity of 100% (95% CI 63–100%) and specificity of 91% (95% CI 75–98%) for the presence of clot in the proximal PAs. An example WIA in patients with and without proximal clot is shown in Fig. 4.

The AUC for AT was 0.84 (95% CI 0.70–0.90). An AT threshold of <57.6 ms was associated with a sensitivity of 88% (95% CI 47–99%) and specificity of 81% (95% CI 64–93%) for the presence of proximal clot.

### Steady Flow Hemodynamics and WIA

There was no observed association between steady hemodynamic parameters and BCW_mean_ area, acceleration time, or PWV (Table 3).

**Table 3.** Simple linear correlations between WIA metrics and acceleration time with hemodynamic and clinical variables

	FCW_mean_ Area, cm^5^		BCW_mean_ Area, cm^5^		FCW_mean_ Peak Time, ms		BCW_mean_ Peak Time, ms		Acceleration Time_mean_, ms		PWV_mean_, m/s	
	*R*	*P*	*R*	*P*	*R*	*P*	*R*	*P*	*R*	*P*	*R*	*P*
PVR	−0.66*	0.001*	−0.13	0.59	−0.44	0.05	−0.44	0.05	−0.41	0.07	0.004	0.96
TPG	−0.56*	0.01*	0.21	0.93	−0.44	0.05	−0.49*	0.03*	−0.37	0.11	0.09	0.71

* Significant parameters.

There was a strong negative correlation between FCW_mean_ area and TPG (*R* = −0.56, *P* = 0.01) and PVR (*R* = −0.68, *P* = 0.001). Of note, the FCW_mean_ area also correlated significantly with RVEF (*R* = 0.65, *P* = 0.002)

The BCW_mean_ peak time was negatively associated with TPG (*R* = −0.49, *P* = 0.03).

## DISCUSSION

In this proof of concept study, we have demonstrated for the first time the feasibility of performing noninvasive WIA in the PAs using phase contrast MR imaging. The main findings were as follows: *1*) there was a significant difference in WIA metrics between patients and controls; *2*) the presence of a BCW was specifically associated with the presence of PH; and *3*) the magnitude of the BCW area showed discriminatory capacity for the presence of proximal PA clot in patients with CTEPH. We believe that these preliminary results demonstrate that WIA could be used in the noninvasive assessment of patients with PH.

Semiquantitative studies of wave reflection have been attempted before in PH. However, most have been limited to assessment of either pressure or flow. For example, in CTEPH, a “notch index” on PA Doppler velocity profiles and shorter acceleration time are associated with greater in-hospital mortality and persistent postoperative PH (8). Unfortunately, pressure-only measures such as inflection time or augmentation index have been shown to be less reliable markers in PH (2, 19). The benefit of WIA is that it includes both flow and a proxy measure of pressure, thereby providing a more accurate assessment of wave reflections.

Using WIA, we demonstrated a marked difference in early backward wave reflections (BCWs in patients and BEWs in normal subjects). This is consistent with the experimental animal work of Hollander et al. (9) in which BEWs were present in normal canine PAs and BCWs in experimentally vasoconstricted PAs. These findings can be attributed to the different types of reflecting sites found in the two models. In normal PAs, the increasing total vessel area at each bifurcation results in reflection sites with predominately negative reflection coefficients and consequent backward expansion waves. Conversely, the reduced vessel area and increased stiffness found in the vasoconstricted state creates reflection sites with positive reflection coefficients and results in compressive reflections. Our data are also concordant with studies of the ovine fetal pulmonary circulation, where large BCWs were observed in the setting of high in utero PVR (33). Unfortunately, there are a limited number of human studies in the pulmonary circulation. An invasive WIA study by Lau et al. (16) did report the presence of a BCW in PH, although small BCWs were also observed in the controls. This is probably because hemodynamic measurements were made in the more distal pulmonary lobe branches to obtain a stable catheter position. The greater proximity to the terminal branches likely explains the presence of BCWs in normal controls.

Interestingly, in our study the reflection site of the BCW was ∼2–3 cm from the site of measurement in the branch PAs (based on wave timing and PWV). Thus quantifiable reflections in the branch PAs seem to arise from the next generation of vessels rather than the terminal branches. This initially appears surprising as the majority of vascular remodeling in PH occurs in the peripheral PAs. However, recent work in the systemic vasculature has demonstrated that wave reflections do not arise from a single discrete reflecting site, rather they are an amalgamation of reflections, with more proximal arising waves being exponentially more important (3). This so-called “horizon effect” is due to re-reflection and entrapment of reflected waves and may be even more important in the highly fractal pulmonary circulation. This effect may also explain the lack of correlation between steady state hemodynamics and BCW, as they reflect different attributes of the vasculature.

Clinically, the presence of an early BCW in the branch PAs could be used as a supplementary method of identifying patients with PH. It may be particularly useful in situations where diagnostic indictors such as septal curvature (23) are less reliable (i.e., patients with PH related to complex congenital heart disease). Nevertheless, this was not a diagnostic cohort study and further work must be performed to evaluate true diagnostic accuracy.

Another finding that may have clinical utility was that BCW area was able to discriminate between patients with proximal CTEPH and those with other forms of PH. This was in spite of the fact that there were no invasive hemodynamic differences between the two groups. Combined with the lack of correlation between steady-state hemodynamics and BCW, this reiterates the fact that BCW provides “novel” hemodynamic information. This novel information could be used as the basis for a noninvasive and nonionizing test for patients with treatable proximal clot. Of course, a larger comparative study would be required to show that it has benefit over current MR perfusion methods (21). Another intriguing possibility is that this new information may provide additional prognostic information over conventional hemodynamic measures. This cannot be predicted from this small study and warrants further investigation.

Two final issues that arise from our results are related to acceleration time and RV function. Our data confirm that abnormal wave reflections explain the shorter acceleration time (13, 14, 30) and notched/scalloped flow/velocity curves (8) observed in PH. Specifically, notching of the flow curve in PH (the point of measurement of AT) occurs when the abnormally large BCW exceeds the incident FCW. Shortened acceleration times can therefore be considered an epiphenomenon of disease-associated wave reflection. It is for this reason we believe that AT performs less well than the WIA components. Regarding RV function, we demonstrated that FCW was lower in patients with PH and correlated significantly with RVEF. It has previously been shown that peak aortic FCW is proportional to LV (max dP/d*t*)^2^ (20) and responds to alterations in the inotropic state of the ventricle (11, 25). Thus FCW may provide another alternative method of assessing RV function in PH. However, significantly more work is required to investigate whether it has any benefits over RVEF.

An important aspect of this study was the use of high temporal resolution area and flow data for the calculation of PWV. This was achieved using a respiratory self-navigated, cardiac gated, golden-angle spiral PCMR sequence. This sequence has the benefit of being able to acquire data at a 10.5-ms temporal resolution, while also maintaining edge sharpness. However, it should be noted that PWVs reported in this study were significantly lower than previously published measures. This is particularly true of studies that used the conventional pressure-velocity (PU) technique (12). For instance, PA PWV in animals has been reported to be between 2 and 3 m/s (5, 9), while Lau et al. (16) reported a PWV of 3.8 m/s in controls vs. 6.9 m/s in patients with PH. This can be partly explained by early reflections that contaminate the period during which PWV is measured. Specifically, it has recently been shown that in the presence of positive reflections the QA-method underestimates PWV, while the PU method overestimates PWV (32). Importantly, the PU method overestimates PWV to a greater extent than the QA method underestimates it (appendix 1). Such overestimation with the PU method may be further exacerbated if PWV is calculated over a longer time period. This is pertinent as several previous studies using the PU method relied on sampling periods of up to 100 ms (compared with 30 ms in this study).

Interestingly, our BCW timing data suggest that reflections occur earlier in PH than in normal controls and in some cases are present in the first 30 ms of the cardiac cycle. When using the QA method this results in greater underestimation of PWV in PH compared with controls (in whom reflections occur later in ejection). Thus with the use of our method it may be more difficult to detect increased PWV in patients with PH. Nevertheless, we believe this more conservative approach is preferable to methods that might overestimate PWV, as it is less likely to artificially conflate group differences. However, studies using the QA method have also reported higher PWV. For instance, Ibrahim et al. (10) reported values 2–3 m/s in cardiac patients without PH and 5.2 m/s in those with PH. These differences can be probably be explained in two ways. Firstly, previous studies have used sequences with lower “true” temporal resolution, which is known to result in overestimation of PWV (27). Secondly, imaging the pulmonary trunk (rather than the branch PAs) may result in overestimation of PWV. This is because as the main PA moves inferiorly in systole, the narrower distal portion moves into the imaging plane, resulting in underestimation of ΔA and increased PWV. Unfortunately, the absence of a “reference standard” method of assessing PWV in the pulmonary vasculature makes it difficult to fully assess the validity of our technique. Nevertheless, we did compare our PWVs with a novel method developed for use in the coronary arteries (4) in the presence of significant reflections and showed good agreement (appendix 2).

### Limitations

The main limitation of this study was the fact that only three points were used to calculate PWV. This was done to limit contamination of the area and flow curves with reflections, which as previously mentioned can have significant effects on the measurement of PWV. However, it may result in increased susceptibility to errors, as the measured PWV is very sensitive to inaccuracies in any of the three measurements.

The purpose of this study was to demonstrate the feasibility of noninvasive WIA and characterize differences between patients and controls. The significant finding of a BCW only present in PH patients suggests utility as a potential diagnostic test. However, a diagnostic testing cohort would be necessary to confirm this.

The role of BCW area for the detection of clot similarly shows potential as an adjunct to noninvasive PH assessment. However, the modest sample size necessitates further work, as reflected in the confidence intervals for sensitivity and specificity.

In this study, cardiac catheterization data and CMR data were not simultaneously acquired; however, the intervening period between catheterization and CMR was short, and patients did not have any significant clinical changes between studies. Catheter based calculations of flow were also made from thermodilution. We cannot exclude the possibility that given simultaneous catheter and CMR assessment there would be stronger correlations with hemodynamics.

This technique does not presuppose a particular pressure-area relationship, and therefore, the units of noninvasive WIA in this study (m^5^/s) do not have an easily understandable physical meaning: in contrast to the units of invasive WIA (W/m^2^). However, the waveforms produced by noninvasive WIA are qualitatively similar to invasive WIA in the literature and given a linear pressure-area relationship would be proportional. Area and flow waves can therefore be considered analogous to pressure and velocity waves as found in the WIA literature.

## CONCLUSION

In conclusion, we have shown that noninvasive pulmonary WIA reveals important abnormalities in patients with PH, distinguishing the disease state from normality, and shows potential as a biomarker to identify PH and differentiate PH subtypes.

## GRANTS

We acknowledge the support received from the British Heart Foundation and UK National Institute of Health Research. This report is independent research by the National Institute for Health Research Biomedical Research Centre Funding Scheme. The views expressed in this publication are those of the authors and not necessarily those of the National Health Services, National Institute for Health Research, or the Department of Health.

## DISCLOSURES

No conflicts of interest, financial or otherwise, are declared by the author(s).

## AUTHOR CONTRIBUTIONS

Author contributions: M.A.Q., J.A.S., L.T., P.S., and V.M. conception and design of research; M.A.Q. and D.S.K. performed experiments; M.A.Q. and V.M. analyzed data; M.A.Q., S.M., A.M.T., P.S., J.G.C., and V.M. interpreted results of experiments; M.A.Q. and V.M. prepared figures; M.A.Q. and V.M. drafted manuscript; M.A.Q., D.S.K., J.A.S., L.T., S.M., A.M.T., P.S., J.G.C., and V.M. edited and revised manuscript; M.A.Q., D.S.K., J.A.S., L.T., S.M., A.M.T., P.S., J.G.C., and V.M. approved final version of manuscript.

## Figures and Tables

**Fig. A1.**
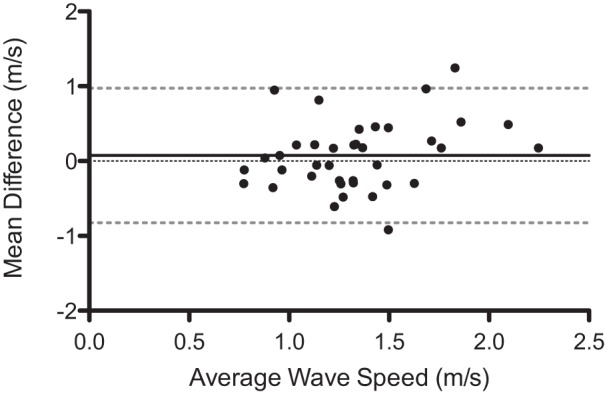
Bland-Altman of bias (black solid line) and 95% limits of agreement (gray broken lines). Data are presented as difference between flow-area (QA) method and minimization of net wave energy method (*y*-axis) vs. the average of both (*x*-axis).

## Appendix 1

In presence of a positive reflection, γ, the PU loop overestimates with a proportionality factor (1 + γ)/(1 − γ), while the QA-loop method underestimates with (1 − γ)/(1 + γ). For a value of γ = 0.3, for instance, the PU-loop method overestimates by 86%, while the QA-loop method underestimates by 46%.

## Appendix 2

In the absence of a “reference standard” method for measuring PWV in the pulmonary vasculature, we compared the PWV estimated using the QA method with a novel method developed by Davies et al. (4) for use in the coronary arteries in the presence of significant reflections. This method works by minimizing net wave energies and was designed to use simultaneously acquired pressure (P) and velocity data (U) from a single position within a vessel. It does not require the vessel to be long enough for two measurements nor does it rely on a period during which there is only a single wave impulse. The method can be modified for use with flow and area measurements:

(9) $$c=\sqrt{\frac{\sum dA^{2}}{\sum dQ^{2}}}$$

With the use of Bland-Altman analysis, we observed good agreement between the two methods, bias of 0.07 m/s (standard deviation of bias, 0.45 m/s) and 95% limits of agreement −0.83–0.97 m/s (Fig. A1).
